# Heterogeneity of outcomes in randomized controlled trials on implant prosthodontic therapy is hindering comparative effectiveness research: meta-research study

**DOI:** 10.1186/s12903-023-03658-9

**Published:** 2023-11-22

**Authors:** Ante Vardić, Livia Puljak, Tea Galić, Joško Viskić, Ena Kuliš, Tina Poklepović Peričić

**Affiliations:** 1 Study of Dental Medicine, University of Split School of Medicine, Split, Croatia; 2 Center for Evidence-Based Medicine and Health Care, Catholic University of Croatia, Zagreb, Croatia; 3 Department of Prosthodontics, Study of Dental Medicine, University of Split School of Medicine, Split, Croatia; 4 Department of Fixed Prosthodontics, University of Zagreb School of Dental Medicine, Zagreb, Croatia

**Keywords:** Core outcomes, Implant prosthodontics, Randomized controlled trials, Meta-research

## Abstract

**Background:**

Consistency in outcomes across clinical trials allows for comparing and combining results from different studies. A core outcome set (COS), representing a minimally agreed standardized group of outcomes that should be monitored and measured through research in a specific field of medicine, is not yet available for trials in implant prosthodontic (dental implant) therapy. This meta-research study aimed to analyze outcomes used in clinical trials on implant prosthodontic therapy.

**Methods:**

We searched the Cochrane Oral Health Group (COHG) register to identify systematic reviews of interventions in implant prosthodontic therapy published by October 2023. From the randomized controlled trials (RCTs) included in the relevant reviews, we extracted data on the characteristics of the included trials and the outcomes used. We categorized outcomes into domains.

**Results:**

From 182 systematic reviews in the COHG register, we included 11 systematic reviews on dental implant therapy. The reviews included 117 unique RCTs with 4725 participants, published from 1995 to 2020, which analyzed 74 different outcomes. Using different definitions, implant failure was analyzed in 73 RCTs. Seventeen RCTs did not define implant failure. Failure was most often (30 RCTs) followed up for one year. Only one RCT assessed implant failure after five years. Trials used 17 definitions of implant failure, while 17 trials did not report on the criteria of implant failure. Complications were analyzed in 48 RCTs, although they were not clearly defined in 12 RCTs. Failure of prosthodontic supra-structure was analyzed in 74 RCTs, with definitions of failure and criteria not clearly defined in 44 RCTs. Trials considered adverse events, peri-implant tissue health, patient attitudes, and other outcomes, including cost, aesthetics, or procedure duration. These outcomes were often different between trials. Twenty-six outcomes were used only once per study.

**Conclusions:**

Clinical trials in implant prosthodontics used different outcomes, different definitions of outcomes and used different times to monitor them. Standardization of outcomes is necessary to allow comparability and evidence synthesis about the effectiveness of implant prosthodontic therapy.

**Supplementary Information:**

The online version contains supplementary material available at 10.1186/s12903-023-03658-9.

## Background

Implant prosthodontics includes treatment planning and restoration of dental implants to replace a lost tooth. Dental implants can restore masticatory function and improve quality of life [1–3], and have therefore become a popular treatment option for partially or completely edentulous patients [4]. However, despite the high success rate and low percentage of complications of such therapy, some patients experience dental implant failure [5].

Depending on when it occurs, implant failure may be classified as early or late, i.e. before or after functional implant loading [5]. Early failure represents the inability to establish osseointegration and includes biological complications affecting the hard and soft tissues surrounding the implant [6–8]. Occlusal forces during chewing, swallowing and biting or during lateral movements of the jaw significantly affect the dental implant-retained prosthetis [9]. Late failure is defined as the inability to maintain the established osseointegration and implant function. It is accompanied by biological and mechanical complications, such as fracture of the implant body, screw body, or implant supra-structure [5, 6, 10].

However, there is no single clear definition of the criteria for assessing the failure of implant therapy [11].

Criteria to assess the success of dental implant therapy have changed over time. The most frequently considered criteria have been survival of dental implants, stability of prosthesis, radiographic evidence of bone loss, and the absence of infection of the peri-implant tissues [12–14]. Based on the available criteria, dental implant therapy is considered successful if there is no mobility of the implant at the start of the prosthetic phase, bone loss is less than 0.2 mm per year after the first year, there is no radiolucency around the implant, there are no signs of peri-implantitis with suppuration, and no symptoms of pain, neuropathy or nerve paraesthesia [12, 15, 16].

The appearance of the soft tissues surrounding the implant, assessment of prosthodontic supra-structure, aesthetics and patient satisfaction were also proposed as criteria of success [17–19].

Heterogeneity in defining the success and failure of dental treatment therapy in clinical practice indicates the possibility that clinical studies may use different outcomes [12–14]. Furthermore, inconsistent outcomes across studies make it impossible to directly compare and systematically summarize all available evidence by combining results from various studies [12, 15, 17]. Thus, it is crucial to standardize outcomes and outcome measures in research on dental implant therapy.

Ideally, a core outcome set (COS) should be used, representing a minimally agreed standardized group of outcomes that should be monitored and measured through research in a specific field of medicine to enable comparison and combination of the results from different studies [20]. However, in the field of dental implant therapy, there is currently no COS, and so far, no studies have examined all outcomes used in clinical trials on implant prosthodontic treatment.

Therefore, this study aimed to map all the outcomes assessed in clinical studies about the efficacy of dental implant therapy and analyze outcomes used to define treatment success or failure of dental implants.

## Materials and methods

### Study protocol

We developed a protocol for this study before the study started. The protocol was not published; it is available in Supplementary file 1.

### Study design

We conducted a cross-sectional meta-research study of randomized controlled trials (RCTs) included in published systematic reviews (SRs) from the Cochrane Oral Health Group (COHG) register. The COHG utilizes Cochrane Oral Health’s Trials Register, which conducts regular searches in the databases CENTRAL, MEDLINE and Embase.

### Eligibility criteria

We analyzed RCTs included in SRs of interventions assessing the effectiveness of different treatment approaches to dental implant therapy, including different implant techniques, different sizes and shapes of implants, or different times for implant loading. We also included SRs that analyzed preoperative therapy before implantation.

### Search

We used the COHG register of published SRs (available at: https://oralhealth.cochrane.org/oral-health-evidence) to identify eligible SRs. The following search terms were used: “dental implants“[All Fields] OR “dental implants“[MeSH Terms] OR „dental implant“[Text Word] OR „oral implantation“[Text Word] OR „oral implanting“[Text Word] OR „dental implanting“[Text Word].

We screened 182 published SRs from the COHG register, which covered a wide range of dental medicine topics. The search was first conducted on May 27th 2022 and then updated on October 21st 2023.

### Screening

Two authors (AV, EK) independently screened titles and abstracts of all SRs from the COHG list of reviews in the first screening phase. Subsequently, in the second screening phase, two authors (AV, EK) independently assessed full texts of potentially eligible SRs for inclusion. Disagreements about including full texts were resolved by discussion between the two authors (AV, EK) or by consulting the senior author (TPP).

### Data extraction

Initially, full texts of RCTs included in the eligible SRs were obtained. A data extraction form was developed for this study and was first piloted on three randomly selected SRs. After final refinements to the extraction form, one author (AV) extracted the data, and another (EK) verified the extractions. The following data were extracted: title of the SR, first author of the SR, publication year of the SR, the list of all included RCTs, first author of the RCT, publication year, number of participants, participants’ health status, the country in which the RCT was conducted, and the list of all outcomes as they were reported in the Results section of each RCT.

Additionally, from each RCT, we extracted information concerning outcomes related to the implant or prosthetic supra-structure, including definitions of the implant or prosthetic failure and follow-up times, and the type of prosthodontic supra-structure used (fixed or removable). Also, data were extracted on outcomes related to the status of the tissues surrounding the implant, as well as specific outcomes considered as postoperative complications and adverse events. All other outcomes, such as those related to how patients accept dental implants, clinicians’ preferences towards a specific procedure, evaluation of aesthetics, and difficulty or duration of the procedure, were also extracted and included in analyses.

### Outcome categorization

Outcomes were categorized according to their similar features into the following domains: outcomes related to the implant itself or the prosthetic supra-structure, outcomes related to complications and adverse events, outcomes related to peri-implant tissue health, and patient-related outcomes. The domain concerning the health of the peri-implant tissues was further subcategorized into oral hygiene outcomes, outcomes related to soft tissue, and bone-related outcomes. Finally, the remaining outcomes that did not fit in any of the previous four domains were categorized as “other outcomes”, e.g. cost of therapy, treatment time, etc.

The decision on categorizing domains was based on the discussion among authors (AV, TG, JV, TPP). Data were entered into a Microsoft Office Excel spreadsheet and appropriately coded.

### Data analysis

We used descriptive summary statistics with absolute numbers and percentages to present the number of studies in each SR, the number of study participants, and the frequency of each outcome across the studies. Data were analyzed using MedCalc, version 19.4 (MedCalc Software, Ostend, Belgium).

## Results

### General characteristics of included studies

Among the 182 screened SRs, 168 records that did not fit the inclusion criteria during the first screening phase were excluded. The remaining 14 SRs were reviews that were in any kind of way associated with the implant prosthodontic therapy. However, after analyzing the full text of those 14 SRs, we excluded three more SRs, of which two were excluded because dental implant therapy for replacing missing teeth was not the intervention of interest. One of the mentioned reviews instead assessed mini-implants’ use for orthodontic anchorage [21], while the other review assessed the effects of various interventions, hyperbaric oxygen therapy and antibiotics, for preventing osteoradionecrosis in the jaws of patients treated for head and neck cancer before implant placement [22]. The third review was excluded [23] because it did not include a single RCT, i.e. it was an empty review.

Finally, 11 SRs [24–34] were included, which were published between 2009 and 2021. The selected SRs included a total of 118 RCTs. Among the 118 RCTs, there was one overlapping trial [35]. Thus, we analyzed 117 unique RCTs, which were published from 1995 to 2020.

**Fig. 1:**
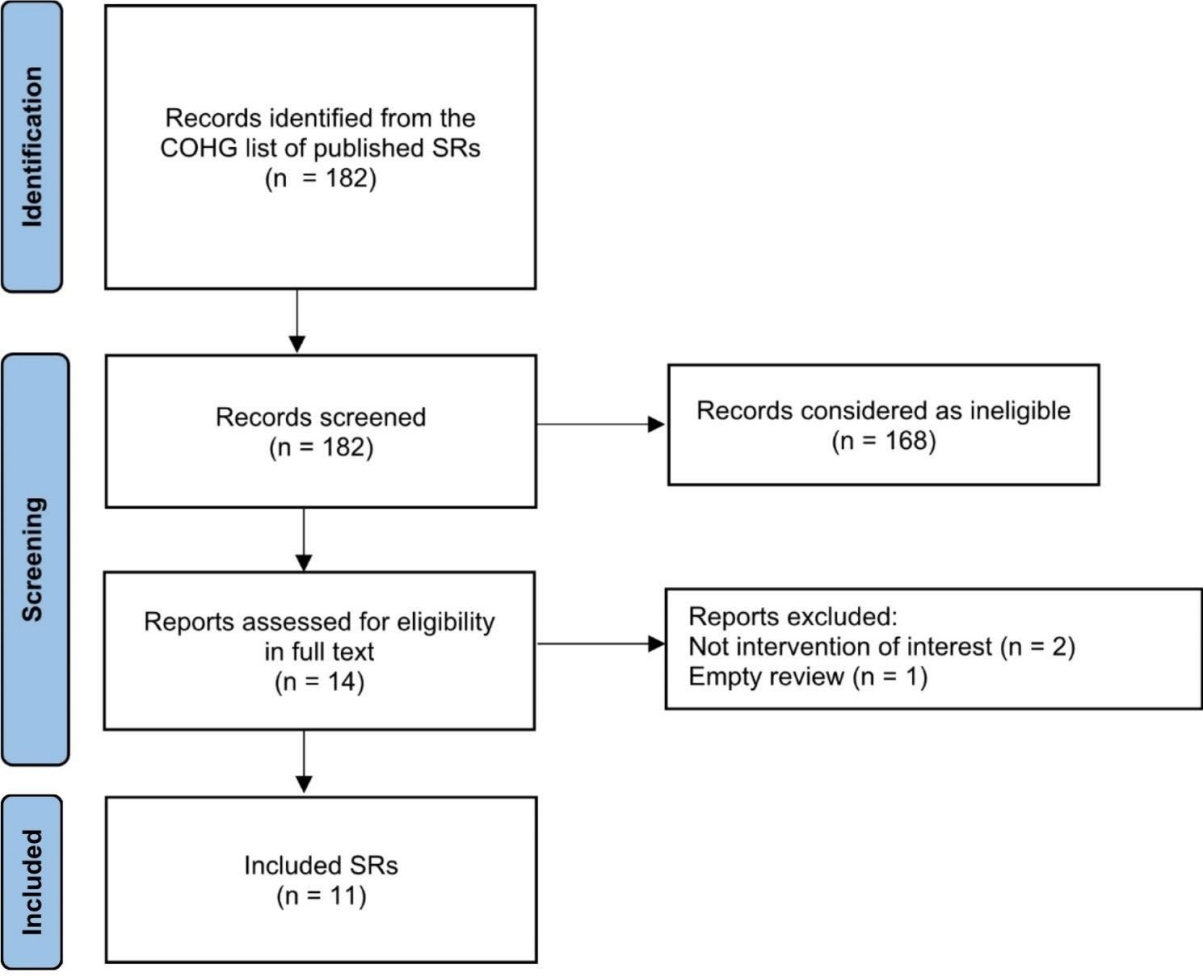
Flow diagram. Acronyms: COHG = Cochrane Oral Health Group; SR = systematic review

We used the PRISMA flowchart to depict the flow of SRs during the searching and screening phase of this study [36]. A list of all included SRs and RCTs is provided in Supplementary file 2. The flow diagram of screening and study inclusion is shown in Fig. 1.

The 117 RCTs were conducted in 27 countries worldwide; most trials (N = 40) took place in Italy. For five trials, it was not reported where the trials were conducted, and one was a multicenter trial reportedly conducted in ten countries, but with no information on which countries. The list of countries with the number of trials conducted in each country is available in Supplementary file 3.

The 117 RCTs included a total of 4725 participants (range: from 5 to 496 participants). In 92 RCTs, the participants were healthy individuals; those studies excluded participants whose health conditions could affect the success of the therapy or cause a deviation in the results. In two RCTs, participants had an unspecified periodontal disease but were otherwise healthy. In one RCT, all participants had head or neck cancer in their medical history and were treated with radiotherapy. One study included participants using long-term non-steroidal anti-inflammatory drugs (NSAID) therapy, requiring antibiotic prophylaxis before surgical procedures. 22 RCTs did not specify the health condition of the subjects.

Supplementary file 4 consists of raw data extracted from the 117 RCTs.

### Analysis of outcomes

A total of 74 different outcomes were used in 117 RCTs. Six RCTs [37–42] assessed only one outcome, and four RCTs [43–46] assessed just two outcomes. Outcomes were categorized into five main domains, as well as ‘other outcomes’.

### Outcomes related to the implant itself or prosthetic supra-structure

We identified seven outcomes related to the implant itself or prosthetic supra-structure (Table 1). Implant failure was assessed in 73 different trials [16, 35, 46–116] (Table 1).

**Table 1:** Outcomes related to the implant itself or prosthetic supra-structure

OUTCOME	N of trials
Implant failure	73
Supra-structure failure	74
Implant Stability Quotient (Osstell device)	11
Implant survival	10
Implant success	3
Implant mobility (periotest)	8
Crown success	2

Ten trials [63, 64, 117–124] assessed implant survival, of which only four trials reported on the criteria for defining survival. Si et al. [63] referred to the definitions suggested by Buser in 1997 [14] and Cochran in 2002 [125] that consider implant mobility, pain and discomfort, peri-implant infection, and continuous radiolucency around the implant. According to these definitions, implant loss, mobility or removals in case of progressive marginal bone loss, severe peri-implant infection, or implant fracture were considered implant failure. Torres et al. [64] defined survival as implants remaining in situ during clinical observations, while Patel et al. [124] reported survivals as “any dental implant which remained in situ without signs of mobility”. In the trial by Enkling et al. [119] implant was considered survival if it remained stable when tested by removal resistance. No other information was provided on how this testing was done.

Six trials [118, 119, 121–124] reported data on survival after a one-year follow-up, while three studies al [63, 64, 117] provided a two-year follow-up for survival. Horwitz et al. [120] assessed survival after three months.

Various definitions of implant failure were reported, with as many as 17 different criteria to define failure across trials (Table 2). Most trials used the criteria proposed by Albrektsson et al. in 1986 [11] that consider implant mobility, pain, dysesthesia, peri-implant infection with suppuration, peri-implant radiolucency, and peri-implant bone resorption of more than 1.5 mm in the first year of function and more than 0.2 mm in the following years (Table 2). In 17 trials [35, 67, 68, 80, 86, 95, 97, 101, 104, 105, 108, 110–115], the definition of failure was not provided.

**Table 2:** Criteria of implant failure from 73 trials that used this outcome

DEFINITION CRITERIA (verbatim when possible)	N of trials
Criteria by Albrektsson et al. 1986	10
“..implant mobility and removal of stable implants dictated by progressive marginal bone loss or infection.”	8
Loss of implant	6
“..presence of any mobility of the individual implant and/or any infection.”	6
Implant mobility	5
Lack of osseointegration	5
Mobility and/or any situation dictating removal	3
“..implant mobility and removal of stable implants dictated by progressive marginal bone loss, infection or implant fracture.”	3
“..lack of implant stability, presence of radiolucent zone around the implants, mucosal suppuration and pain.”	2
“..implant mobility, removal of stable implants dictated by progressive marginal bone loss or infection, and any mechanical complications (e.g. implant fracture) rendering the implant unusable. Also, implants that had to be removed at implant insertion due to lack of stability and the risk of falling into the sinus cavity.”	1
“implant mobility, removal of stable implants as a result of progressive bone loss and implant fracture”	1
“A failed implant was defined as the presence of signs of infection and/or radiographic peri-implant radiolucencies that could not respond to a course of antibiotics and/or judged a failure after performing an explorative flap surgery by an experienced periodontologist.”	1
Mobility, bone loss, radiolucency, pain, discomfort and/or neurosensory alteration	1
“..fracture, mobility when tested, peri-implant radiolucency, pain, discomfort, infection, and/or marginal bone loss that could not be alleviated by clinical intervention.”	1
“..radiolucency around the implant, mobility and suppuration, pain or ongoing pathologic processes.”	1
“..implant mobility; presence of peri-implant radiolucency, recurrent peri-implant infection, continuous or recurrent pain, or structural failure of the implant; and > 0.2 mm bone resorption between any two visits.”	1
“..progressive peri-implant loss of bone that exceeds the limits of tolerable bone absorption after successful osseointegration.”	1
Definition not reported	17

Furthermore, trials used 13 different follow-up times to assess the failure of implant therapy (Table 3). The most commonly used follow-up time was 12 months, followed by 24 months. Only one trial [52] provided data after a five-year follow-up, which was recommended for assessing implant failure by Albrektsson et al. in 1986 [11]. Almost a quarter of trials (23%) followed-up implant failure for more than 12 months (Table 3).

**Table 3:** Follow-up time for implant failure in 73 trials that used this outcome

FOLLOW-UP TIME	N of trials
3 months	2
3–4 months	2
4 months	3
5 months	2
6 months	10
9 months	1
1 year (12 months)	30
18 months	2
19 months	1
2 years (24 months)	11
3 years (36 months)	7
4 years (48 months)	1
5 years (60 months)*	**1**

*recommended follow-up time for implant failure (by Albrektsson at al., 1986)

Among 74 trials that assessed prosthesis failure [35, 43–46, 49, 50, 52–99, 101–106, 117–119, 121–123, 126–131], two types of prosthetic supra-structure were used: fixed (screwed or cemented) (N = 46; 62%) and removable (overdenture) (N = 14; 18%). In eight (11%) trials [49, 50, 63, 64, 101, 104–106], the type of prosthetic supra-structure was not reported. Different definitions of prosthesis failure outcome were used in 30 trials [43–45, 49, 50, 52–60, 62, 72–74, 77, 79, 81, 82, 87, 92, 93, 98, 102, 128–130], but mostly considered prosthesis failure as the inability to place planned prosthesis due to implant failure or loss of prosthesis following implant failure [49, 50, 52, 53, 55–59, 62, 72–74, 92, 93].

Hall et al. [81] assessed the failure of fixed prosthetic supra-structures using criteria proposed by Walton [55], and two other trials published by Felice et al. in 2009 [56, 57] evaluated the success of mandibular overdentures using the six-step protocol proposed by Payne [58]. The criteria to assess prosthodontic success for implant retained overdentures referred to in these trials [56, 57, 81] consider the following: patrix and matrix loosening, fracture, number of times they were activated or replaced, fracture of implant overdenture, the need to reline or construct a new overdenture, and peri-implant or inter-abutment mucosae enlargement. These criteria were first included in a classification protocol proposed by Walton in 1998 [55]. In 2002 Payne [58] published a protocol for prosthodontic maintenance, including the same specific categories. Therefore, we considered these criteria as one outcome in our analyses. The trials [56, 57, 81] that assessed prosthodontic maintenance using general categorization according to either the classification protocol proposed by Walton [55], or the Payne protocol [58] were therefore presented together.

A total of 44 trials [35, 61, 63–71, 75–78, 80, 16, 83–86, 88–91, 94–97, 99, 101, 103–106, 117–119, 121–123, 126–128] out of 74 trials did not report any details about defining this outcome (Table 4).

**Table 4:** Definitions of prosthesis failure from 74 trials that used this outcome

DEFINITION		N of trials
Prosthesis that could not be placed due to implant failure, or loss of prosthesis secondary to implant failure		15
Prosthodontic maintenance by general categorization, including criteria by Walton 1998 and Payne 2002s		7
Prosthesis could not be placed due to implant failure, or loss of prosthesis secondary to implant failure, or any prosthesis in need of replacement		2
Fracture or component failure		3
Prosthesis mobility		2
Need for adjustments of repair		1
Definition not reported		**44**

### Outcomes related to postoperative complications and adverse events

From 117 RCTs, only 14 trials [47–51, 107, 108, 110–115, 132] referred to outcomes related to adverse events. Postoperative pain [51] and postoperative infection [132] were assessed as adverse events in one trial each. A set of outcomes, including erythema multiforme, urticaria, nausea, vomiting, diarrhoea etc., were assessed in two trials [49, 50]. However, most trials (N = 10) reported on “adverse events” using a general term without specifying any details about the outcome.

Postoperative complications were assessed in 48 trials [35, 48–51, 53–62, 64, 65, 69, 72–75, 82, 87, 92–94, 96, 98, 99, 101, 102, 104–108, 110–114, 119, 122, 123, 127, 128, 133], but they used different sets of outcomes to assess complications (Table 5). Most trials (N = 16) [53, 55–60, 62, 72–75, 92, 93, 106, 133] considered any prosthetic or biological complications like wound or implant infection, mucositis, abscesses or periimplantitis. One trial [96] reported complications as “any biologic complications”, while some trials assessed more specific outcomes, like edema, erythema, wound dehiscence, inflammation etc.

**Table 5:** Outcomes reported as postoperative complications

OUTCOME (Verbatim where appropriate)	N of trials
Any prosthetic or biological complications	16
Postoperative pain or any kind of discomfort	5
Peri-implant mucositis or peri-implantitis	2
“..wound dehiscence, suppuration, fistula, abscess, osteomyelitis, etc.”	2
Any biologic complications	1
Not specified	12
“..internal and external edema, internal and external erythema, pain, heat, and exudate.”	1
“Inflammation, redness of the mucosa, wound dehiscence, sequestration, and loss of bone particles.”	1
Sinus membrane dehiscence	1
“…such as unexpected deviations from the normal treatment outcome; examples of biological complications are haemorrhaging during and after implant placement and/or peri-implantitis.”	1
“Minor” or “major” complications based on the criteria established by Enislidis et al.	1
“..inflammation, wound infection, wound dehiscence, sensory disturbances of lip and chin.”	1
“..pain, fatigue; bowel function, breathing, appetite and sleep disorders.”	1
Swelling	1
Allergic reactions, swellings, abscesses or infections	1
“Post-operative swelling, bruising, suppuration and wound dehiscence”	1

Two trials [102, 108] specified complications as peri-implant mucositis and peri-implantitis. Criteria proposed by Enislidis [134] were used in one trial [94], while other trials (N = 16) [48–51, 54, 64, 65, 82, 98, 99, 101, 105, 113, 114, 122, 123] included outcomes like dehiscence, occlusal interference, tilting of segment, pain, swelling, hypesthesia, fracture of the basal bone, breakage of distractor, infection, inflammation, or breakage either in terms of mechanical block preventing distractor activation, instability of distractor or disengagement of a threaded rod from the basal stabilizing plate, as well as fracture of transport segment.

The remaining 12 trials [35, 61, 69, 87, 104, 107, 110–112, 119, 127, 128] did not specify any details about complications.

Eleven trials [48–51, 107, 108, 110–114] considered both types of outcomes, postoperative complications and adverse events. However, there were some overlaps between these two groups of outcomes. Namely, postoperative pain and infection were considered postoperative complications in some trials [54, 99, 101, 105], while in others, they were regarded as adverse events [51, 132].

### Indicators of peri-implant tissues health

Trials used various outcomes to assess the status of the tissues surrounding implants which we categorized into three subdomains: outcomes related to oral hygiene, outcomes related to soft tissue, and bone-related outcomes (Table 6). The plaque index was most often (N = 22) [54, 61, 70, 79, 80, 83, 99, 100, 105, 110, 115, 116, 119, 123, 135–141] used to assess the level of oral hygiene around implants. There were 16 different outcomes related to the soft tissue surrounding implants, of which the most common outcomes were “probing pocket depth” (N = 37) [46, 54, 61, 69–71, 76, 78, 80, 83, 84, 99, 100, 103, 105, 107–115, 118, 119, 123, 124, 128, 133, 135, 138, 139, 141–143], and “bleeding on probing index” (N = 28) [46, 54, 61, 76, 99, 103, 105, 107–116, 118, 119, 123, 133, 135, 136, 138, 134, 141–143]. Trials used 12 different bone-related outcomes. The most commonly used outcomes were “Radiographic peri-implant marginal bone level changes” (N = 32) [46, 60–62, 66, 69–71, 73, 74, 84–88, 90, 91, 99, 106, 118, 121, 123, 127, 128, 143], “height and width of the alveolar ridge” (N = 15) [37–42, 84, 105, 106, 116, 124, 129, 131, 144, 145] and “peri-implant marginal bone levels” (N = 14) [52, 55–59, 63, 75, 92, 93, 108, 119, 128, 133]. We extracted data regarding the type of radiographic imaging used to evaluate peri-implant marginal bone level changes and found that most trials (N = 20) [46, 69–71, 73, 74, 84–88, 106, 118, 127, 128] reported using standardized intraoral imaging. Periapical imaging was used in eight trials [60–62, 66, 77, 90, 91, 121], while panoramic [76, 143] and extra-oral oblique lateral imaging [99, 123] were used in two trials each.

**Table 6:** Indicators of peri-implant tissue health

SUBDOMAIN	OUTCOME	N of trials
OUTCOMES RELATED TO ORAL HYGIENE	Plaque index	22
	Plaque accumulation	16
	Calculus (yes/no)	3
	Lobene stain index	2
OUTCOMES RELATED to SOFT TISSUE	Probing pocket depth	37
	Bleeding on probing index	28
	Gingival recession	10
	Gingival index	9
	Sulcus bleeding index	8
	Clinical “attachment” level	8
	Width of the keratinized mucosa	8
	Microbiological evaluation	7
	Mucosa level	5
	Soft tissue thickness	3
	Papilla index	3
	Papilla levels	2
	Bleeding time index	1
	Crevicular fluid flow rate	1
	Soft tissue volumetric analysis	1
	Thickness of buccal wall	1
BONE-RELATED OUTCOMES	Radiographic peri-implant marginal bone level changes	32
	Height and width of alveolar ridge	15
	Peri-implant marginal bone levels	14
	Vertical bone gain after augmentation	11
	Peri-implant marginal bone level changes	7
	Radiographic bone gain	4
	Bone gain maintenance over time	1
	Bone level changes after loading	1
	Bone to implant distance	1
	Vertical distraction distance	1
	Bone width at implant site	1
	Maxillary bone width changes over time	1

### Patient-related outcomes

Trials used eight different patient-related outcomes (Table 7). Patient satisfaction (N = 19) [43–45, 56, 58, 69, 72–74, 99, 101, 106, 119–121, 123, 128, 130, 131] and patient preference (N = 9) [53–57, 92, 102, 106, 130] were used most commonly across trials.

**Table 7:** Patient-related outcomes

OUTCOME	N of trials
Patient satisfaction	19
Patient preference	9
Prosthetics acceptance	2
Patient compliance	1
Anxiety during treatment	1
Functional assessment and quality of life	1
Subjective chewing ability	1
Subjective evaluation of taste and change in taste	1

### Other outcomes

The remaining outcomes could not be categorized into any of the previous groups and were categorized as “other outcomes” (Table 8). Histomorphometric evaluation (N = 13) [35, 61, 64, 65, 92, 95, 97, 98, 105, 106, 126, 131, 144] was the most commonly used outcome across trials. It represents the histological evaluation of specimens of bone tissue to assess either newly formed bone tissue, bone graft material, loose connective tissue, quality of the bone, or the presence of bone resorption, etc. Only one trial provided no information about the process of histomorphometric evaluation [98], while the remaining 12 trials reported how the process was carried out.

**Table 8:** Other outcomes

OUTCOME	N of trials
Histomorphometric evaluation	13
Aesthetics	10
Preoperative augmentation failure	8
Duration of the operative procedure	7
Need for additional augmentation	4
Treatment time (defined as the time from the start of preoperative procedures to the placement of prosthetic supra-structure)	3
Cost of therapy	2
Clinician preference	2
Implant percussion	1
Days needed to start the prosthetic rehabilitation	1
Difficulty of the procedure (in a technical sense)	1

Aesthetics was assessed in ten trials [56, 68, 69, 105, 106, 117, 118, 121, 127, 128], of which in three RCTs [56, 117, 127], aesthetics was evaluated by the dentists, and in one trial [118] it was evaluated by patients. In three trials [68, 121, 128], aesthetics was evaluated by both dentists and patients. One RCT [69] used an independent blinded evaluator. Two RCTs [105, 106] used objective evaluation using validated indices to assess aesthetics. One trial [105] used colour blending of the grafted site with the adjacent soft tissues and the other trial [106] used pink aesthetic scores (PES). Two trials assessed clinicians’ preferences that refer to either operator’s preferences regarding different techniques applied at implant placement [53], or preferences towards augmentation procedures before implant placement [57].

## Discussion

This meta-research study found considerable heterogeneity in outcomes used in RCTs on implant prosthodontics. Overall, 74 different outcomes were used in 117 RCTs, of which 24 were used in one trial only. Trials used a wide range of different criteria to define the outcomes. Most trials provided no information on the criteria used to define these specific outcomes. Furthermore, trials measured outcomes at very different times.

With the wide use of implants to replace lost teeth [1], there is a parallel increase in the number and the extent of research in the field of dental implant therapy [146]. However, our study indicates the need for improvement in the consistency and usability of outcomes used in the field. The most commonly assessed outcomes included implant and prosthesis failure, postoperative complications, adverse events, and implant survival. For implant failure, the most commonly used outcome, 17 different definitions were used. Researchers used 13 different follow-up times for implant failure, most commonly one year period, reported in 30 studies. Only one trial analyzed implant failure at five years, which is the recommended minimum time for monitoring the success rate of implant prosthetic therapy according to the criteria of Albrektsson et al. [11].

The short-term follow-up can be understood as a pragmatic choice of trialists. RCTs are complex studies requiring intense resources – time-consuming, expensive, and often burdensome on patients [147]. However, an inadequate trial follow-up period contributes to a lack of reliable evidence for decision-makers [148]. Long-term data collection and analysis are crucial when evaluating a procedure such as dental implant placement [149].

Furthermore, adequate outcomes need to be chosen to evaluate the success/ failure of dental implants properly. Considering the rate of complications in implant-supported fixed partial dentures (FPD) after five years, it is important to include prosthesis success in analyses of the overall success of dental implants [150]. Namely, studies should evaluate a long-term primary outcome by considering the implant prosthetic complex as a whole [151].

In our study, survival, prosthetic failure, and implant failure were assessed separately in many studies. A systematic review by Papaspyridakos et al., which was published in 2012 [151]., addressed success criteria in RCTs and prospective studies on implant dentistry published from 1980 to 2010. They pointed out that in the dental implant literature, survival/success rates of single parameters were often presented, but that single parameters used as success criteria should be regarded as surrogate endpoints, as they are often used to compensate for the lack of well-defined primary outcomes. They advised that, for example, bone loss or any other outcome alone within an implant prosthodontic rehabilitation should not be considered as the measure of success. Papaspyridakos et al. argued that current advances in contemporary implant prosthodontics, coupled with high patients’ expectations, necessitate a more comprehensive definition of success criteria for implant/prosthodontic procedures. They suggested that future studies should choose outcomes that reflect the complexity of the implant-prosthetic complex and consider multiple outcomes as measures of success or implant failure [151].

Papaspyridakos et al. found similar results to ours, with different trials using different criteria to assess the success of the implant therapy. Furthermore, they found that the reported success rate of dental implant therapy consistently decreased when the number of parameters included for measuring success was increased [151].

In 2009, Gallucci et al. proposed success criteria for implant-supported fixed complete dental prostheses (FCDPs) based on the implant, peri-implant tissues, prosthodontic, and subjective parameters. They suggested that complete dental prostheses (CDPs) were deemed as successful when a total of four or fewer complications (mild or moderate severity) occured and when these could be addressed chair-side in a single visit [152].

Our study showed that patient satisfaction was analyzed in only 19 of 117 trials (16%). According to Levi et al., patient satisfaction with overall treatment should be rated good or excellent for the treatment outcome to be considered successful [153].

Furthermore, a conceptual framework proposed for understanding the outcomes of dental implant therapy also includes psychological outcomes related to the patient and economic aspects. However, in our sample, only two trials assessed the cost of treatment [154].

Aesthetics is also an important outcome in dental implant treatment for both patients and clinicians [155]. The Pink Esthetic Score (PES) and the White Esthetic Score (WES) have been proposed for measuring aesthetics in implant prosthodontics [16, 18]. In this study, only ten trials assessed aesthetics. However, only two trials used validated scales to assess aesthetics, the colour blending of the grafted site with the adjacent soft tissues and the PES, while eight trials did not explain in detail how they assessed aesthetics at all. Those studies only mentioned that aesthetics was assessed by the clinician or patient.

Problems with outcome heterogeneity, different definitions, and lack of definitions were noted in other research fields as well. For example, a systematic review found ten different definitions for postoperative mortality in esophageal cancer research, most of which were not clearly described, and there were different interpretations of the term in-hospital mortality [156, 157].

Heterogeneity in outcomes measured across studies in the same disease or treatment hamper clinical evaluation, trial comparability, and effective evidence synthesis [157]. The results of heterogeneous studies cannot be combined, compared in systematic reviews, and further used for developing clinical practice guidelines. Therefore, their applicability in the context of other research and clinical practice remains questionable, including their contribution to overall scientific knowledge and clinical practice as well.

For example, 24 out of 74 outcomes found in analyzed trials were used once per trial. A meta-analysis of such outcomes is not possible. In addition, another 14 outcomes were measured in five or fewer primary studies, meaning that overall more than half (38 of 74) of the outcomes were replicated in more than 5% of studies. If we were to look only at outcomes that were repeated in more than 10% of the primary studies, we would have only 13 such outcomes. A deleterious example of single use of outcomes was reported in a newly published study from the field of oncology that found as many as 25,000 different outcomes used only once in oncology trials [158].

Furthermore, for clinically meaningful outcomes, all clinical trials of interventions must analyze not only efficacy but also harms. In our study, only 14 trials out of 114 analyzed adverse events.

Adverse events are less likely to be reported than efficacy outcomes, and different methods of assessing adverse effects produce different reported incidences [159, 160], despite available reporting guidelines suggesting the inclusion of adverse events in research papers [161, 162]. Golder et al. found that a median of 43% of published studies reported adverse events data, compared with a median of 83% of unpublished studies. A wider range of specific adverse events was found in sources other than published journal articles. In addition, when published and unpublished reports of the same study were compared, it was shown that the unpublished version was more likely to contain adverse effects data (median 95%) compared with the published version (median 46%) [163].

Postoperative complications were defined differently in included trials, most often as any kind of prosthetic or biological complications. A systematic review indicated that biological and technical complications following dental implant therapy should be better specified [164].

This study included only RCTs included in available Cochrane SRs, which may be regarded as a limitation. It is acknowledged that there could be relevant RCTs that were not included in the analyzed SRs. However, considering the comprehensive search and robust methodology of Cochrane SRs, as well as the wide period during which the trials analyzed in our study were published (from 1995 to 2020), it is highly likely that these reviews covered clinical questions of highest priority and included all relevant clinical trials concerning implant prosthodontics. Also, we made arbitrary decisions when designing this study, because there are no methodological guidelines for conducting analyses of outcomes. Most importantly, increasing the sample of studies in this work is unlikely to change the results. We have already proven on this sample that there is a very high heterogeneity of methodological approaches in the studied field.

This study can be helpful for the next step of developing a COS for dental implant therapy. The development and application of an agreed COS have been spearheaded by the COMET (Core Outcome Measures in Effectiveness Trials) Initiative, which was launched in 2010 [19]. The COS is mainly developed by relevant experts in a particular field of medicine, but it also involves the public to ensure that those outcomes are relevant and important to patients. If the findings are to influence policy and practice, then the chosen outcomes need to be relevant and important to key stakeholders, including patients and the public, health care professionals, and others making decisions about health care [165].

When searching the literature, we found no similar research that took a methodological look at the diversity of outcomes in research on dental implant prosthetics.

The COS in the field of implant prosthodontics has not been proposed yet. Therefore, our results have the potential to contribute to the development of COS and improve the use of relevant outcomes in future trials on implant prosthodontic therapy. The use of COS would lead to a standardized use of outcomes, enable future synthesis and comparison of the obtained results within different studies and preserve resources for the improvement of future research.

The creation and use of COS in studies leads to a reduction in the number of studies that are considered research waste, which is defined as studies that are not necessary, and have poor design, conduct or reporting. Research waste hinders or prevents the synthesis and usability of study results [166, 167].

## Conclusions

Clinical trials in implant prosthodontics used different outcomes, different definitions of outcomes and used different times to monitor them. Standardization of outcomes is necessary to allow comparability and evidence synthesis about the effectiveness of implant prosthodontic therapy. Future research should include defining a core outcome set for implant prosthodontic therapy.

### Electronic supplementary material

Below is the link to the electronic supplementary material.

Supplementary Material 1: Study protocol

Supplementary Material 2: List of included systematic reviews and randomized controlled trials

Supplementary Material 3: Countries in which randomized controlled trials were conducted

Supplementary Material 4: Raw data extractions

## Data Availability

Raw data generated by extractions from the included trials and all relevant data concerning SRs or RCTs included in our analyses are available in Supplementary file 4.

## List of abbreviations

CDP: Complete dental prostheses
COHG: Cochrane Oral Health Group
COMET: Core Outcome Measures in Effectiveness Trials Initiative
COS: Core outcome set
FCPD: Fixed complete dental prostheses
FPD: Fixed partial dentures
NSAID: Non-steroidal anti-inflammatory drugs
PES: Pink Aesthetic Scores
RCT: Randomized controlled trial
STROBE: STrengthening the Reporting of OBservational studies in Epidemiology
WES: White Esthetic Score
