# Supplementary material for: Heterogeneity of outcomes in randomized controlled trials on implant prosthodontic therapy is hindering comparative effectiveness research: meta-research study
Source: BMC Oral Health. 2023 Nov 22;23:908. doi: 10.1186/s12903-023-03658-9 (PMC10666438; doi:10.1186/s12903-023-03658-9)
Supplement: Supplementary file 1 — Supplementary Material 1: Study protocol [file 12903_2023_3658_MOESM1_ESM.docx]

**Analysis of outcomes used in randomized controlled trials about efficacy and safety of implant prosthodontic therapy: a protocol for a meta-research study**

Ante Vardić^1^, Livia Puljak^2^, Tea Galić^3^, Joško Viskić^4^, Ena Kuliš^1^, Tina Poklepović Peričić^3^

^1^ Study of Dental Medicine, University of Split School of Medicine, Split, Croatia

^2^ Center for Evidence-Based Medicine and Health Care, Catholic University of Croatia, Zagreb, Croatia

^3^ Department of Prosthodontics, Study of Dental Medicine, University of Split School of Medicine, Split, Croatia.

^4^ Department of Fixed Prosthodontics, University of Zagreb School of Dental Medicine, Zagreb, Croatia

**Keywords:** *core outcomes*, *implantology*, *randomized controlled trials*, *meta-research*

**Background**

Dental implants now stand as an indispensable part of clinical dentistry for replacing missing teeth [1]. Because of their ability to restore masticatory function and improve quality of life [2,3], dental implants have become a popular treatment option for partially or completely edentulous patients [4]. However, despite the high success rate and low percentage of complications of such treatment, some patients experience dental implant failure [5].

Criteria to assess the success of dental implants changed over time, and have most often considered survival of dental implants, stability of prosthesis, radiographic evidence of bone loss, and the absence of infection of the peri-implant tissues [6,7,8]. Various criteria for defining success of implant therapy have been proposed, for example lack of mobility of the implant at the start of the prosthetic phase, bone loss after the first year is less than 0.2 mm per year, there is no radiolucency around the implant, no peri-implantitis with suppuration, and no symptoms of pain, neuropathy or nerve paraesthesia [6,9,10].

The appearance of the soft tissues surrounding the implant, assessment of prosthodontic supra-structure, aesthetics and patient satisfaction have also been proposed as success criteria [11,12,13].

Heterogeneity in defining the success and failure of dental treatment therapy in clinical studies may makes it impossible to directly compare and systematically summarize all results from the available studies [14,15].

Ideally, a core outcome set (COS), representing a minimally agreed standardized group of outcomes that should be monitored and measured through research in a specific field of medicine should be used, to enable comparison and combination of the results from different studies [16].

Unfortunately, in the field of dental implant therapy, there is no COS currently, and no studies have examined all outcomes used in dental implantology clinical trials conducted thus far.

Therefore, this study aimed to map all the outcomes assessed in clinical studies about the efficacy of dental implant therapy and to analyse outcomes used to define treatment success or failure of dental implants.

**Materials and methods**

***Study design***

We will conduct a cross-sectional meta-research study of randomized controlled trials (RCTs) included in published systematic reviews (SRs). The study will be reported in accordance with the **STrengthening the Reporting of OBservational studies in Epidemiology** (STROBE) guidance [17].

***Eligibility criteria***

We will analyze RCTs included in SRs of interventions assessing the effectiveness of different treatment approaches to dental implant treatment, including different implant techniques, different sizes and shapes of implants, or different times for implant loading. We will also include SRs that analyzed preoperative treatment prior to implantation.

***Search***

We will use the Cochrane Oral Health Group (COHG) register of published SRs (available at: https://oralhealth.cochrane.org/oral-health-evidence) to identify eligible SRs.

***Screening***

Two authors will independently screen titles and abstracts of all SRs from the COHG list of reviews in the first screening phase. Subsequently, in the second screening phase, they will independently assess full texts of potentially eligible SRs for inclusion. Disagreements about the inclusion of full texts will be resolved by discussion or by consulting the senior author.

***Data extraction***

We will obtain full texts of RCTs included in the eligible SRs. A data extraction form will be developed for this study and will be piloted on three randomly selected SRs. One author will extract the data, and another author will verify the extractions. The following data will be extracted: title of the SR, first author of the SR, publication year of the SR, the list of all included RCTs, first author of the RCT, publication year, number of participants, participants’ health status, a country in which the RCT was conducted, and the list of all outcomes, including outcomes related to the implant itself or prosthetic supra-structure, outcomes related to the status of the tissues surrounding the implant, as well as specific outcomes concerning postoperative complications and adverse events, and all other outcomes as they are reported in the Results section.

***Data analysis***

Extracted data will be entered into an Excel spreadsheet and appropriately coded.

We will use descriptive summary statistics with absolute numbers and percentages to present the number of studies in each SR, the number of participants in studies, and the frequency of each outcome across studies. Data will be analyzed using MedCalc, version 19.4 (MedCalc Software, Ostend, Belgium).

**References:**

1. Alghamdi HS, Jansen JA. The development and future of dental implants. Dent Mater J. 2020 Mar 31;39(2):167-172.
2. Hartlev J, Kohberg P, Ahlmann S, Andersen NT, Schou S, Isidor F. Patient satisfaction and esthetic outcome after immediate placement and provisionalization of single-tooth implants involving a definitive individual abutment. Clin Oral Implants Res. 2014 Nov;25(11):1245-1250.
3. Jofre J, Castiglioni X, Lobos CA. Influence of minimally invasive implant-retained overdenture on patients' quality of life: a randomized clinical trial. Clin Oral Implants Res. 2013 Oct;24(10):1173-7.
4. Shemtov-Yona K, Rittel D. An Overview of the Mechanical Integrity of Dental Implants. Biomed Res Int. 2015;2015:547384.
5. Esposito M, Hirsch JM, Lekholm U, Thomsen P. Biological factors contributing to failures of osseointegrated oral implants. (I). Success criteria and epidemiology. Eur J Oral Sci. 1998 Feb;106(1):527-51.
6. Albrektsson T, Zarb G, Worthington P, Eriksson AR. The long-term efficacy of currently used dental implants: a review and proposed criteria of success. Int J Oral Maxillofac Implants. 1986 Summer;1(1):11-25.
7. Misch CE, Perel ML, Wang HL, Sammartino G, Galindo-Moreno P, Trisi P, Steigmann M, Rebaudi A, Palti A, Pikos MA, Schwartz-Arad D, Choukroun J, Gutierrez-Perez JL, Marenzi G, Valavanis DK. Implant success, survival, and failure: the International Congress of Oral Implantologists (ICOI) Pisa Consensus Conference. Implant Dent. 2008 Mar;17(1):5-15.
8. Annibali S, Bignozzi I, La Monaca G, Cristalli MP. Usefulness of the aesthetic result as a success criterion for implant therapy: a review. Clin Implant Dent Relat Res. 2012 Mar;14(1):3-40.
9. Buser D, Mericske-Stern R, Bernard JP, Behneke A, Behneke N, Hirt HP, Belser UC, Lang NP. Long-term evaluation of non-submerged ITI implants. Part 1: 8-year life table analysis of a prospective multi-center study with 2359 implants. Clin Oral Implants Res. 1997 Jun;8(3):161-72.
10. Lindeboom JA, Frenken JW, Dubois L, Frank M, Abbink I, Kroon FH. Immediate loading versus immediate provisionalization of maxillary single-tooth replacements: a prospective randomized study with BioComp implants. J Oral Maxillofac Surg. 2006 Jun;64(6):936-42.
11. Fürhauser R, Florescu D, Benesch T, Haas R, Mailath G, Watzek G. Evaluation of soft tissue around single-tooth implant crowns: the pink esthetic score. Clin Oral Implants Res. 2005 Dec;16(6):639-44.
12. Meijer HJ, Stellingsma K, Meijndert L, Raghoebar GM. A new index for rating aesthetics of implant-supported single crowns and adjacent soft tissues--the Implant Crown Aesthetic Index. Clin Oral Implants Res. 2005 Dec;16(6):645-9.
13. Belser UC, Grütter L, Vailati F, Bornstein MM, Weber HP, Buser D. Outcome evaluation of early placed maxillary anterior single-tooth implants using objective esthetic criteria: a cross-sectional, retrospective study in 45 patients with a 2- to 4-year follow-up using pink and white esthetic scores. J Periodontol. 2009 Jan;80(1):140-51.
14. Chalmers I, Glasziou P. Avoidable waste in the production and reporting of research evidence. The Lancet. 2009 Jul 4;374(9683):86-9.
15. Williamson PR, Altman DG, Blazeby JM, Clarke M, Devane D, Gargon E, Tugwell P. Developing core outcome sets for clinical trials: issues to consider. Trials. 2012 Dec;13(1):1-8.
16. COMET (Core Outcome Measures in Effectiveness Trials) Initiative. <http://www.comet-initiative.org/>.
17. **The Strengthening the Reporting of Observational Studies in Epidemiology (STROBE)statement: guidelines for reporting observational studies.**J Clin Epidemiol. 2008 Apr;61(4):344-9.
