# Supplementary material for: Heterogeneity of outcomes in randomized controlled trials on implant prosthodontic therapy is hindering comparative effectiveness research: meta-research study
Source: BMC Oral Health. 2023 Nov 22;23:908. doi: 10.1186/s12903-023-03658-9 (PMC10666438; doi:10.1186/s12903-023-03658-9)
Supplement: Supplementary file 3 — Supplementary Material 3: Countries in which randomized controlled trials were conducted [file 12903_2023_3658_MOESM3_ESM.docx]

**Supplementary file 3. Countries in which the randomized controlled trials were conducted**

| Country | N of RCTs |
| --- | --- |
| Italy | 40 |
| Netherlands | 14 |
| Germany | 6 |
| USA | 6 |
| Sweden | 7 |
| Spain | 5 |
| New Zealand | 4 |
| Belgium | 3 |
| China | 3 |
| Australia | 2 |
| Denmark | 2 |
| Germany and Switzerland | 2 |
| Turkey | 2 |
| Austria | 1 |
| Brazil | 1 |
| Canada | 1 |
| Colombia | 1 |
| Egypt | 1 |
| Germany and USA | 1 |
| India | 1 |
| Iran | 1 |
| Ireland | 1 |
| Israel | 1 |
| Portugal | 1 |
| Romania | 1 |
| Serbia | 1 |
| South Korea | 1 |
| United Kingdom | 1 |
